# The hidden microbiome of hospital infection surveillance testing: biomarkers of health outcomes in MRSA and VRE colonization

**DOI:** 10.21203/rs.3.rs-3299277/v1

**Published:** 2023-08-31

**Authors:** Bashir Hamidi, Lisa L. Steed, Scott R. Curry, Cassandra D. Salgado, Alexander V. Alekseyenko

**Affiliations:** Medical University of South Carolina

**Keywords:** Methicillin-resistant Staphylococcus aureus, vancomycin-resistant Enterococcus (VRE), microbiome predictor of health, colonization resistance, translational research, 16S rRNA gene

## Abstract

**Background:**

Hospital-acquired infections present a major concern for healthcare systems in the U.S. and worldwide. Drug-resistant infections result in increased costs and prolonged hospital stays. Methicillin-resistant *Staphylococcus aureus* (MRSA) and vancomycin-resistant *Enterococcus* (VRE) are responsible for many drug-resistant infections in the U.S. We undertook two parallel studies aimed to investigate the differences in the microbial communities of individuals colonized with MRSA (or VRE) as compared to their respective non-colonized counterparts matched for age, sex, race, ethnicity, unit of admission, and diagnostic-related group, when available.

**Results:**

The VRE study showed considerably more *Enterococcus* genus communities in the VRE colonized samples. Our findings for both MRSA and VRE studies suggest a strong association between 16S rRNA gene alpha diversity, beta diversity, and colonization status. When we assessed the colonized microbial communities in isolation, the differences disappeared, suggesting that the colonized microbial communities drove the change. Isolating *Staphylococcus,* we saw significant differences expressed across colonization in specific sequence variants.

**Conclusions:**

The differences seen in the microbial communities from MRSA (or VRE) colonized samples as compared to non-colonized match-pairs are driven by the isolated communities of *the Staphylococcus* (or *Enterococcus)* genus, the removal of which results in the disappearance of any differences in the diversity observed across the match-pairs.

## BACKGROUND AND SIGNIFICANCE

Programs of infection prevention for pathogens such as methicillin-resistant *Staphylococcus aureus* (MRSA) and vancomycin-resistant *Enterococcus* (VRE) are routine in many hospitals in the United States due to the associated risks to patients. MRSA alone accounts for over 94,000 annual infections and almost 19,000 deaths, with 86% of the cases associated with healthcare [1–4]. Moreover, healthy individuals in non-healthcare settings are susceptible to community-acquired MRSA infections. Both within healthcare and community settings, the majority of people colonized with MRSA are asymptomatic, and unless regular and routine infection surveillance testing is performed, transmission and acquisition may go unrecognized. Infections due to these multidrug-resistant bacteria are associated with increased rates of morbidity, mortality, and healthcare costs, making them a major public health concern [5]. It is also known that colonization with drug-resistant bacteria increases the risk for subsequent infection [6–8].

Understanding the factors contributing to the colonization and persistence of MRSA and VRE in human populations is crucial for the development of effective prevention and control strategies. Prior to 2020, the Medical University of South Carolina (MUSC) utilized an active surveillance testing program designed to identify patients colonized with MRSA and VRE and exercise the use of contact precautions for those found to be colonized. Under this program, a majority of patients admitted for over 24 hours are tested for MRSA using nasal swabs, producing approximately 15,000 specimen tests for the pathogen annually. High-risk hospitalized patients undergo active surveillance testing for MRSA and VRE weekly until the patient becomes positive for MRSA, VRE, or is discharged.

Investigating the microbiome’s role in MRSA and VRE colonization may shed light on the complex interactions between bacteria and their human hosts. There are important scientific and clinical implications for our research. Firstly, by characterizing the nasal and perianal microbiome in MRSA and VRE colonization, we aim to identify microbial signatures that could serve as potential signatures for colonization risk. These biomarkers may help identify those who are more likely to become colonized and may facilitate the implementation of focused preventative therapies to limit the spread of antibiotic-resistant pathogens in healthcare settings. Secondly, understanding the variations in microbial diversity and community structure among individuals colonized and uncolonized may provide insight into the mechanisms underlying antibiotic resistance. Studies such as ours could help elucidate how specific microbial species or groups interact with MRSA/VRE and affect the likelihood of colonization. This may lead to the development of novel interventions aimed at specific microbial communities to reduce colonization and subsequent infection rates.

## MATERIALS AND METHODS

### Study population

We utilized infrastructure from the Living μBiomeBank to enroll, process, sequence, and perform downstream analyses [9]. The MUSC Institutional Review Board protocol (Pro00062584) was approved as a minimal-risk study, using existing surplus nasal and perianal swab specimens processed by the MUSC Diagnostic Microbiology Laboratory. Specimens from pediatric populations and those who have opted out of participating in clinical research from discarded clinical specimens via intake questionnaire were excluded.

Two parallel studies were undertaken to assess the differences in the microbial communities of those colonized with MRSA and VRE as compared to their uncolonized matched counterparts. The methods describe the parallel and similar processes for enrolling specimens from these populations and downstream handling and processing. Daily reports using the electronic medical health record platform Epic identified nasal specimens, reported MRSA/VRE status, demographic variables, admission unit, admission diagnosis, and other identifiers. MRSA/VRE positive specimens were captured on an ongoing basis, de-identified, and stored at −80°C for batch processing. Once a MRSA/VRE positive specimen was identified, a one-to-one matched MRSA/VRE negative specimen was sought based on age (± 5 years), sex, race, ethnicity, unit of admission, and diagnostic-related group, when available. To minimize potential biases due to seasonal microbiome variability, matching MRSA/VRE negative samples were at most sought within four weeks of the original MRSA/VRE positive specimens’ collection dates. If a match was not had within the prespecified window, the positive specimen was discarded, and another positive specimen was recruited.

### DNA extraction and 16S rRNA gene sequencing

Samples were processed for bacterial DNA extraction using QIAamp DNA Mini Kit from Qiagen (Hilden, Germany) according to the manufacturer’s instructions. The quality and purity of extracts were determined using the QIAxpert system (Qiagen). Bacterial 16S rRNA variable region V2 was amplified with Illumina (San Diego, CA, USA) Nextera XT Index Kit v2 index adapters (CTGTCTCTTATACACATCT) [10, 11]. Barcoded libraries were pooled and sequenced on a MiSeq using paired-end sequencing with 2 × 300 cycles. Sequences were then transferred to the Program for Human Microbiome Research at MUSC for further processing and analysis.

### Statistical analysis

All statistical analysis have been performed in R (v. 4.0.3) [12]. Initial preprocessing of the Illumina paired-end sequences was performed using the statistical denoising algorithm DADA2 (v. 1.19.1) [13, 14]; chimeric sequences were identified and removed using the UCHIME algorithm within the DADA2 package [15]; taxonomy was assigned against the SILVA reference database (v. 138.1) [16] using an implementation of the naїve Bayesian classifier similar to the RDP-II classifier [17]. The resulting amplicon sequence variants (ASVs) with corresponding taxonomic information and sample data were transformed into a phyloseq (v. 1.27.6) [18, 19] object for downstream statistical analysis.

A flowchart of the downstream analysis is presented as Supplementary Figure S1. Three analysis scenarios were considered: (a) in which all microbial ASVs are present; (b) in which all ASVs identified as genus *Staphylococcus* or *Enterococcus* have been removed; and (c) in which all ASVs except those classified as genus *Staphylococcus* or *Enterococcus* have been removed. Within each of these three scenarios, figures were generated and analytical tests performed including univariate and multivariate testing. Non-bacterial ASVs were removed as an initial step. Compositional bar charts, alpha diversity figures, and statistical assessments of alpha diversity (paired *t*-tests) were performed prior to any filtering. Filtering threshold was set at to retain ASVs with at least a 1% relative abundance and observed in more than one sample. Following filtering, univariate and multivariate analysis were performed. Relative abundance data were transformed and normalized using centered log-ratio (CLR) calculations to use the geometric mean of the filtered sample vector as the reference. Multiple clustering methods were utilized: Jensen-Shannon divergence and Bray distance matrices utilized relative abundance data, whereas the Euclidean distance was used with the CLR-transformed abundances. Match-stratified multivariate data were analyzed using $W_{d}^{*}$ test, a robust distance-based multivariate analysis of variance which has been developed in our group to account for multivariate dispersion in the data tested, which has been shown to be associated with adverse statistical properties in PERMANOVA [20]. ICD9 and ICD10 codes were extracted and encoded into the Elixhauser Comorbidity Index using python package pyelixhauser [21]. Gap statistic (K-means) clustering analysis was performed on these the Elixhauser comorbidities using R package cluster (v. 2.1.4) [22] to assess the presence of comorbidity clusters in cases and controls for the MRSA and VRE studies. Our study targeted a convenience sample of 50 matched pairs of nasal and 50 matched pairs of perianal swabs.

## RESULTS

Samples (N = 108) were successfully sequenced and generated all downstream data. Tables 1a and b present the demographic summary of the patient population for MRSA and VRE studies, respectively. Controls were recruited by matching on the demographic and comorbidities of cases (sex, age, race, ethnicity, unit of admission, and diagnostic-related group, when available). As indicated in Tables 1a and b, no statistically significant differences (*t*- and chi-squared tests) were observed in age, race, sex, and Elixhauser Comorbidity Index between the cases and controls for the MRSA and VRE studies.

**Figure 1:**
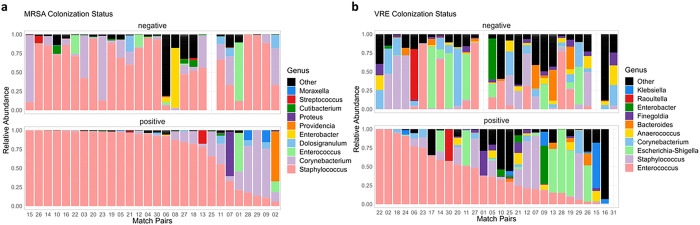
Samples from subjects colonized with MRSA (a, bottom facet), VRE (b, bottom facet), and respective uncolonized match subject pairs (a and b, top facets) are displayed on the x-axes. The relative abundance of the top ten most abundant genus-level taxa are displayed on the y-axis. Less abundant taxa are combined and colored as black (corresponding to “other”). Uncolonized subjects are seen to have higher diversity of bacteria present individually and as a group (number of different taxa not in top 10 genera) as shown in proportional analysis Supplementary Figure S2.

For the MRSA study, 55 samples generated a total of 1,704,344 non-chimeric reads (mean [SD] = 30,988 [12,438.9], median [min, max] = 31,966 [2,298, 72,070]). These comprised 740 ASVs, five (5) of which were eukaryotic in origin and were only observed across four samples. For the VRE study, 53 samples generated a total of 1,648,085 non-chimeric reads (mean [SD] = 31,096 [12,157.35], median [min, max] = 27,840 [8,337, 68,097]). These reads comprised 962 ASVs, only one (1) of which was eukaryotic in origin and was observed across four samples. The eukaryotic ASVs were removed prior to analysis. Sequencing depth between MRSA/VRE colonized and uncolonized samples were assessed to ensure no significant read quality differences were present in any of the covariates. Case-control matched visualization of the top-10 most abundant genus-level communities revealed an interesting trend (Figs. 1a and b); uncolonized samples (top facets) are observed to have a relatively larger portion of their abundance comprise their non-top-10 genera (combined and colored in black), indicating a greater within sample diversity. This trend was confirmed and visualized in Supplementary Figs. 2a and b. As observed in Fig. 1a, the majority of ASVs in both colonized and uncolonized samples were identified within the *Staphylococcus* genus. This same trend was not observed in the VRE study; although in the cases we observe the *Enterococcus* genus with the majority abundance, this is not the case in the matched negative controls (in 1b top facet).

Alpha diversity of samples was assessed (Table 2) prior to any abundance/prevalence filtering under the three scenarios described in the methods. For the MRSA study, a total of 735 ASVs across 55 subjects were assessed using *t*-test of observed, Shannon, and Simpson diversity measures, all three of which were found to be significantly associated with subjects’ colonization status (p = .026, .003, and .004 respectively). ASVs identified as *Staphylococcus* were removed and processed for the same analysis. This time only the observed alpha diversity count was seen to be significant (p = .02). Subsequently, *Staphylococcus* ASVs were isolated and processed using the same assessment of alpha diversity and no significance was observed on a 0.05-level. Similar trends were observed in the VRE study in which observed, Shannon, and Simpson diversity measures were found to be significantly associated with subjects’ colonization status (p = .003, .001, and .012 respectively) when all ASVs were included. ASVs identified as *Enterococcus* were removed and processed for the same analysis leading to significantly different alpha diversity measures again (p = .001, .001, and .003 for observed, Shannon, and Simpson respectively). Subsequently, *Enterococcus* ASVs were isolated and processed using the same assessment of alpha diversity and no significance was observed on a 0.05-level. We have included visualized alpha diversity measures as Supplementary Figs. 3a and b.

A filtering criterion of at least 1% relative abundance and observed in more than one sample was applied. In the MRSA study, this eliminated 566 low abundance and 523 low prevalence ASVs (total of 631), resulting in 104 ASVs that were used for univariate and multivariate assessments. CLR-transformed data was assessed in ASV-wise manner across colonization status (Table 3a). This assessment revealed ASVs 1 and 2 (both identified in the *Staphylococcus* genus) as statistically significant (\varvec*q* = 4.98 × 10^−5^ and 6.32 × 10^−5^, respectively). These two ASVs are also the most abundant overall. When corrected for multiple comparisons using FDR approach, q-values were observed to be non-significant (on a .05 level). A table containing all 104 ASVs is included as Supplementary Table 1a. A similar comparison was performed on genus agglomerated CLR data (Table 4a). We do not see any differences in q-values following corrections for multiple testing. A table containing all 30 genera of the MRSA study is included as Supplementary Table 2a.

We used an identical filtering criterion for the VRE study removing 670 low abundance and 659 low prevalence ASVs (total of 785), resulting in 176 ASVs that were used for our downstream analyses. CLR-transformed data was assessed in ASV-wise manner across VRE colonization status (Table 3b). Although three ASVs (1, 8, and 20) showed significant p-values, when corrected for multiple comparisons using FDR approach, q-values were observed to be non-significant (on a .05 level). ASVs 1 and 8 belong to the *Enterococcus* genus and ASV20 belongs to the *Klebsiella* genus. A table containing all 176 ASVs is included as Supplementary Table 1b. A similar comparison was performed on genus agglomerated CLR data (Table 4b) which showed no significant differences once corrected for multiple comparison. A table containing all 64 genera of the VRE study is included as Supplementary Table 2b.

**Figure 2:**
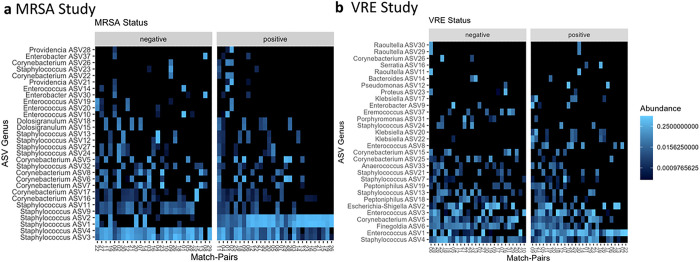
Heatmaps showing constitutive ASV phylotypes across MRSA (a) and VRE (b) colonized and uncolonized respective pairs. Individual samples are shown on the x-axis using the matching pair number. ASVs are shown on the y-axis and labeled using genus-level taxonomy. ASVs and samples are ordered by prevalence. Only the top 30 most abundant ASVs are included within this figure and additional figures with all 104 and 176 observed ASVs, for MRSA and VRE samples respectively, are provided as Supplementary Figures 4 and 5.

The composition of the top 30 most abundant ASVs across samples is displayed using a heatmap in Fig. 2a and b. We see specific ASVs that are associated primarily or exclusively with each group (colonized and non-colonized). In 2a we see *Staphylococcus* ASVs 3 and 4 to be widely present in both cases and controls, whereas ASVs 1 and 2 (also *Staphylococcus*) are almost exclusively observed in the cases (MRSA colonized). On the other hand, *Staphylococcus* ASVs 9 and 11 are observed largely in the controls (non-colonized for MRSA). We have included a larger heatmap including all 104 AVSs within the MRSA study as Supplementary Fig. 4. Assessing these differences across colonization using Pearson’s correlation test shows significance (correlation coefficient = .287, p = .033). In 2b (VRE study) we see *Enterococcus* ASVs 1 and 3 to be primarily present in the cases (VRE colonized), whereas ASV 2 (*Escherichia-Shigella*), ASV 13 (Staphylococcus), and ASV 18 (*Peptoniphilus*) are primarily observed in the controls (non-colonized for VRE). We have included a larger heatmap including all 176 AVSs within the VRE study as Supplementary Fig. 5. Assessing these differences across colonization using Pearson’s correlation test shows no significance (correlation coefficient = .095, p = .49).

**Figure 3:**
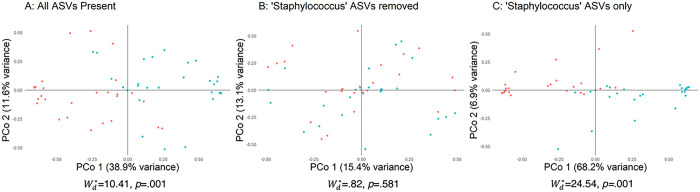
See image above for figure legend

**Figure 4:**
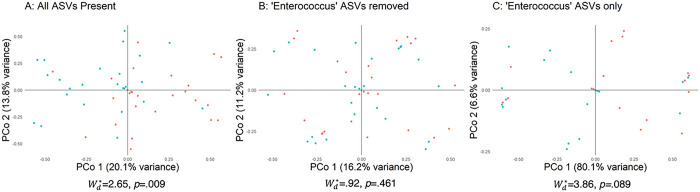
See image above for figure legend

Visualized principal coordinates analysis (PCoA) of the Bray-transformed relative abundance data (β-diversity) revealed interesting interaction of ASVs (Figs. 3 and 4). Here, the assessments were made using the three scenarios (for MRSA and VRE studies) described within the methods section. Statistical assessment was made using $W_{d}^{*}$ test which has been developed in our group to account for heteroscedasticity and control for confounders and covariates [23]. Starting with the MRSA study, when all the ASVs are present (filtering at least 1% relative abundance and seen in more than one sample) (Scenario a, Fig. 3a), we see that the MRSA colonized cases are differentiated from the non-colonizing controls ($W_{d}^{*}=10.41$, p=.001). Removal of the *Staphylococcus* genus (Scenario b, Fig. 3b) results in an overlap of group centroids meaning these differences are primarily driven by the *Staphylococcus* genus, which accounts for most observed microbiome shift ($W_{d}^{*}=.82$, p=.581). Looking at the *Staphylococcus* genus in isolation (Scenario c, Fig. 3c) we see the first axis of PCoA accounting for a large 68.2% of the differences observed ($W_{d}^{*}=24.54$, p = .001), further evidence that the signal results from within this genus. Performing a similar assessment for the VRE study following standard filtering criteria (filtering at least 1% relative abundance and seen in more than one sample), when all the ASVs are present (Scenario a, Fig. 4a), we see that the VRE colonized cases are relatively differentiated from the non-colonizing controls ($W_{d}^{*}=2.65$, p=.009). Removal of the *Enterococcus* genus (Scenario b, Fig. 4b) results in an overlap of group centroids meaning these differences are primarily driven by the *Enterococcus* genus, which accounts for most observed microbiome shift ($W_{d}^{*}=.92$, p=.461). Looking at the *Enterococcus* genus in isolation (Scenario c, Fig. 4c) we see the first axis of PCoA accounting for a large 80.1% of the differences observed ($W_{d}^{*}=3.86$, p=.089). We have performed similar analyses using Jensen-Shannon divergence metric (on relative abundances) and Euclidean distances (on CLR-transformed abundances), along with respective $W_{d}^{*}$ test results for the three aforementioned scenarios. These are included as Supplementary Figs. 6–9 and show similar trends as described above.

## DISCUSSION

The findings in both the MRSA and VRE studies indicated alpha diversity measure differences, notably within the ASVs of genus *Staphylococcus* or *Enterococcus* between the MRSA/VRE colonized and their matched pairs. We assessed these differences further by considering the primary colonizing genus in each study and either removing it entirely or isolating it solely for the analysis. This allowed us to assess the degree to which *Staphylococcus* or *Enterococcus* genera influence the remaining microbial communities of their hosts. In both studies we observe that the alpha diversity of neither *Staphylococcus* nor *Enterococcus* genera are significantly different in isolation whereas the alpha diversity of the remaining community (without *Staphylococcus* or *Enterococcus* genera) is influenced by these two genera. In other words, the inclusion of *Staphylococcus* or *Enterococcus* genera indicate important sources of observed variation in our alpha diversity analysis. Our ASV-level univariate analysis results showed that *Staphylococcus* genus ASV 1 and 2 are significantly different in MRSA colonized vs uncolonized match pairs (paired t-test corrected using FDR). A similar trend was not observed in our VRE study once corrected for multiple testing. Genus-level agglomerated univariate testing also showed no significant differences even in the case of *Staphylococcus* that was previously observed on the ASV-level. This may be indicative of differences in behaviors of species within the same genus such as *Staphylococcus.*

We performed PCoA of the beta-diversity revealing distinct clustering profiles of colonized cases and non-colonized matched controls. A similar pattern is observed in MRSA and VRE studies in which a significant difference is seen when all ASVs are present; this difference disappears entirely when the colonized genus is removed (all ASVs except *Staphylococcus* or *Enterococcus*); and is markedly present in colonized genus in isolation (only *Staphylococcus* or *Enterococcus).* The variance obtained from the PCoA as well as our own robust $W_{d}^{*}$ tests attest to this trend that the differences were primarily driven by the Staphylococcus (or *Enterococcus)* genus.

While the study provides important insights into the compositional microbiome differences of the groups, there are several limitations including the relatively small sample size, which could have made it difficult to detect some of the differences between the two groups. Additionally, the study was conducted in a single hospital, which may limit the generalizability of the findings. The study may also suffer from selection bias given we relied on surplus clinical specimens and there may be inherent biases associated with subjects who are hospitalized or sampled.

The findings from this study shed important light on the variations in the microbiome composition of subjects colonized with MRSA/VRE compared to uncolonized matches. The *Staphylococcus* and *Enterococcus* genera were found to be significant contributors to these differences which may have implications for understanding the colonization dynamics and developing potential interventions for MRSA and VRE infections.

## Figures and Tables

**Table 1:** Comparison of demographic characteristics of enrolled samples from subjects colonized with (a) MRSA and (b) VRE and their respective uncolonized matched subject-pairs (controls). All subjects identified as non-Hispanic/Latino. No statistical significance (at a .05 level) between cases and controls across demographics of the recruited samples were observed.

	a. MRSA Study			b. VRE Study		
	MRSA Cases (N = 28)	Controls (N = 27)	*p*-value*	VRE Cases (N = 27)	Controls (N = 26)	*p*-value*
**Sex**						
Male	13 (46.4%)	13 (48.1%)	1	17 (63.0%)	18 (69.2%)	0.848
Female	15 (53.6%)	14 (51.9%)		10 (37.0%)	8 (30.8%)	
**Age (years)**						
Mean (SD)	56.7 (16.3)	55.6 (16.3)	0.8	57.5 (13.5)	61.0 (11.4)	0.31
Median [Min, Max]	56.0 [25.0, 93.0]	55.0 [25.0, 93.0]		60.0 [20.0, 79.0]	64.0 [38.0, 81.0]	
**Race**						
White	20 (71.4%)	20 (74.1%)	1	21 (77.8%)	20 (76.9%)	1
Black	8 (28.6%)	7 (25.9%)		6 (22.2%)	6 (23.1%)	
**Elixhauser Comorbidity Index**						
Mean (SD)	5.43 (2.50)	5.70 (3.99)	0.762	8.27 (4.13)	7.62 (3.77)	0.554
Median [Min, Max]	6.00 [0, 11.0]	6.00 [0, 13.0]		8.50 [1.00, 16.0]	8.00 [1.00, 14.0]	
Missing				1 (3.7%)	0 (0%)	

* Significance values were calculated using chi-square tests for categorical variables (sex and race) and two-sample t-test for numerical variables (age and Elixhauser Comorbidity Index).

**Table 2.** Assessment of alpha diversity of observed, Shannon, and Simpson indices are assessed using paired t-test (within match analysis) under three scenarios: 1. in which all ASVs present (at least 1% relative abundance and in more than 1 sample); 2. in which all ASVs identified as genus *Staphylococcus* (a) or *Enterococcus* (b) have been removed; and 3. in which all ASVs except those classified as genus Staphylococcus (a) or Enterococcus (b) have been removed. In both (a) and (b), within the first scenario we can see a significant difference (at a 0.05 level) in alpha diversity of observed counts, Shannon and Simpson indices. However, removal of the ASVs of the genus *Staphylococcus* (a) results in the disappearance of this significant difference of the Shannon and Simpson indices previously observed (observed counts remain significant). Likewise, when ASVs of *Staphylococcus* are isolated (a) in assessment, we observe no statistical significance. Removal of the ASVs of the genus *Enterococcus* (b) results in the increase in magnitude of the effect. However, when ASVs of *Enterococcus* are isolated (b) in assessment, we observe a drop in magnitude with no statistical significance.

**a. MRSA Study: Assessment of Alpha-Diversity Measures**								
*Measure*	*Mean*			*Conf Interval*		*df*	*t-statistic*	*p-value*
	*MRSA*−	*MRSA*+	*Delta*	*Low*	*High*			
**All ASVs**								
**Observed**	38.07	27.22	10.85	1.37	20.33	26	2.35	0.026
**Shannon**	2.12	1.63	0.49	0.19	0.80	26	3.31	0.003
**Simpson**	0.79	0.70	0.08	0.03	0.14	26	3.17	0.004
**‘Staphylococcus’ ASVs removed**								
**Observed**	26.96	17.40	10.80	1.82	19.78	24	2.48	0.02
**Shannon**	1.97	1.88	0.16	−0.18	0.51	24	0.98	0.34
**Simpson**	0.74	0.76	0.00	−0.07	0.07	24	0.08	0.94
**‘Staphylococcus’ ASVs only**								
**Observed**	11.54	11.11	0.46	−2.86	3.78	25	0.29	0.78
**Shannon**	1.48	1.31	0.18	−0.08	0.43	25	1.43	0.16
**Simpson**	0.69	0.65	0.04	−0.02	0.10	25	1.31	0.20
**b. VRE Study: Assessment of Alpha-Diversity Measures**								
*Measure*	*Mean*			*Conf Interval*		*df*	*t-statistic*	*p-value*
	*MRSA*−	*MRSA*+	*Delta*	*Low*	*High*			
**All ASVs**								
**Observed**	52.72	28.27	25.62	9.98	41.27	23	3.39	0.003
**Shannon**	2.29	1.58	0.76	0.34	1.18	23	3.70	0.001
**Simpson**	0.76	0.62	0.15	0.04	0.26	23	2.72	0.012
**‘Enterococcus’ ASVs removed**								
**Observed**	50.92	24.85	27.17	11.73	42.61	23	3.64	0.001
**Shannon**	2.42	1.55	0.91	0.44	1.38	23	4.00	0.001
**Simpson**	0.79	0.60	0.20	0.07	0.32	23	3.32	0.003
**‘Enterococcus’ ASVs only**								
**Observed**	2.81	3.42	−0.94	−2.50	0.62	15	−1.28	0.22
**Shannon**	0.31	0.42	−0.15	−0.48	0.17	15	−1.02	0.32
**Simpson**	0.18	0.22	−0.07	−0.25	0.12	15	−0.78	0.45

**Table 3:** ASV-level univariate analysis of centered log-ratio (CLR) abundances on MRSA (a) and VRE (b) colonized and uncolonized respective pairs using paired t-test. Only the top 10 most significant ASVs are displayed here and larger tables including all 104 ASVs (for a) and 176 ASVs (for b) are included as Supplementary Table 1. P-values were corrected for multiple testing using FDR methods (q-values). In (a) we see ASVs 1 and 2 showing a significant q-value (on .05 level) even after these corrections. Both of these ASVs have been identified within the *Staphylococcus* genus and no species level information is available. In (b) we see no individual ASV as statistically significant (on .05 level) after multiple testing corrections.

**a. MRSA Study: ASV-level univariate analysis using paired t-test on CLR-transformed relative abundance values between MRSA colonized and non-colonized matches.**										
*ASV*	*Genus*	*Species*	*Mean*			*Conf Interval*		*t-statistic*	*p-value*	*q-value*
			*MRSA*−	*MRSA*+	*Delta*	*Low*	*High*			
ASV1	*Staphylococcus*	NA	0.03	0.23	−0.21	−0.27	−0.14	−6.64	4.79E-07	4.98E-05
ASV2	*Staphylococcus*	NA	0.03	0.21	−0.18	−0.24	−0.12	−6.27	1.22E-06	6.32E-05
ASV11	*Staphylococcus*	NA	0.01	3.05E-03	0.01	2.93E-03	0.02	2.78	9.88E-03	0.26
ASV4	*Staphylococcus*	NA	0.14	0.07	0.08	0.02	0.14	2.61	0.01	0.26
ASV9	*Staphylococcus*	epidermidis	0.02	4.12E-03	0.01	2.73E-03	0.02	2.61	0.01	0.26
ASV3	*Staphylococcus*	NA	0.17	0.08	0.09	0.02	0.17	2.61	0.01	0.26
ASV8	*Corynebacterium*	NA	0.03	2.81E-03	0.03	7.15E-04	0.06	2.11	0.05	0.5
ASV7	*Corynebacterium*	accolens	0.04	3.98E-03	0.04	−1.01E-03	0.07	2	0.06	0.5
ASV35	*Cutibacterium*	NA	4.84E-03	−3.39E-04	5.25E-03	−1.77E-04	0.01	1.99	0.06	0.5
ASV42	*Cutibacterium*	NA	3.46E-03	−3.59E-04	3.91E-03	−2.04E-04	8.02E-03	1.95	0.06	0.5
**b. VRE Study: ASV-level univariate analysis using paired t-test on CLR-transformed relative abundance values between MRSA colonized and non-colonized matches.**										
*ASV*	*Genus*	*Species*	*Mean*			*Conf Interval*		*t-statistic*	*p-value*	*q-value*
			*VRE*−	*VRE*+	*Delta*	*Low*	*High*			
ASV1	*Enterococcus*	NA	0.07	0.22	−0.16	−0.28	−0.04	−2.73	0.01	0.54
ASV8	*Enterococcus*	faecium	−9.44e-04	0.02	−0.02	−0.04	−5.28e-03	−2.64	0.01	0.54
ASV20	*Klebsiella*	NA	−9.44e-04	8.91e-03	−1.67e-03	−3.30e-03	−4.41e-05	−2.12	0.04	0.54
ASV32	*Anaerococcus*	NA	4.83e-03	6.01e-04	4.89e-03	−1.76e-04	9.96e-03	1.99	0.06	0.54
ASV72	*Anaerococcus*	NA	2.29e-03	3.45e-04	6.78e-04	−3.69e-05	1.39e-03	1.96	0.06	0.54
ASV37	*Eremococcus*	NA	8.15e-03	−9.00e-04	4.43e-03	−4.64e-04	9.33e-03	1.87	0.07	0.54
ASV18	*Peptoniphilus*	NA	9.20e-03	9.68e-04	8.77e-03	−1.09e-03	0.02	1.84	0.08	0.54
ASV124	*Staphylococcus*	lugdunensis	1.49e-03	−7.55e-04	1.88e-03	−2.51e-04	4.01e-03	1.82	0.08	0.54
ASV52	*Murdochiella*	asaccharolytica	4.00e-03	−6.36e-04	4.82e-03	−7.11e-04	0.01	1.80	0.08	0.54
ASV165	*Facklamia*	languida	9.60e-04	−9.00e-04	1.93e-03	−2.88e-04	4.15e-03	1.80	0.09	0.54

**Table 4:** Genus-level univariate analysis of centered log-ratio (CLR) abundances on MRSA (a) and VRE (b) colonized and uncolonized respective pairs using paired t-test. Only the top 10 most significant genera are displayed here and the larger tables including all 30 and 64 genera, for MRSA and VRE samples respectively are included as Supplementary Table 2. P-values were corrected for multiple testing using FDR methods (q-values). In (a) we observe genus *Cutibacterium* on the threshold of significance (on a 0.05 level). However, q-values after multiple testing corrections show no significance. In (b) we observe genera *Enterococcus, Corynebacterium, Mobiluncus,* and *Facklamia* as significance (on a 0.05 level). Likewise here, the q-values after multiple testing corrections show no significance for any genera.

**a. MRSA Study: Genus-level univariate analysis using paired t-test on CLR-transformed relative abundance values between MRSA colonized and non-colonized matches.**								
*Genus*	*Mean*			*Conf Interval*		*t-statistic*	*p-value*	*q-value*
	*MRSA*−	*MRSA*+	*Delta*	*Low*	*High*			
*Cutibacterium*	7.37E-03	−4.83E-03	0.01	−4.57E-04	0.03	1.98	0.058	0.59
*Lawsonella*	−5.32E-03	−6.77E-03	1.42E-03	−3.91E-04	3.24E-03	1.61	0.12	0.59
*Moraxella*	−6.84E-03	3.24E-03	−0.01	−0.02	3.63E-03	−1.52	0.14	0.59
*Enterococcus*	0.03	0.01	0.02	−8.72E-03	0.05	1.4	0.17	0.59
*Staphylococcus*	0.46	0.54	−0.08	−0.2	0.05	−1.28	0.21	0.59
*Enterobacter*	0.02	−6.37E-03	0.03	−0.02	0.07	1.07	0.29	0.59
*Neisseria*	−3.05E-03	−6.68E-03	3.60E-03	−3.46E-03	0.01	1.05	0.3	0.59
*Proteus*	−6.90E-03	9.99E-03	−0.02	−0.05	0.02	−1.02	0.32	0.59
*Providencia*	−6.72E-03	0.01	−0.02	−0.06	0.02	−1	0.33	0.59
*Bacillus*	1.51E-04	−6.52E-03	6.64E-03	−7.76E-03	0.02	0.95	0.35	0.59
**b. VRE Study: Genus-level univariate analysis using paired t-test on CLR-transformed relative abundance values between MRSA colonized and non-colonized matches.**								
*Genus*	*Mean*			*Conf Interval*		*t-statistic*	*p-value*	*q-value*
	*MRSA*−	*MRSA*+	*Delta*	*Low*	*High*			
*Enterococcus*	0.13	0.33	−0.2	−0.33	−0.07	−3.18	0.004	0.26
*Corynebacterium*	0.11	0.02	0.08	0.02	0.13	2.79	0.01	0.32
*Mobiluncus*	−4.78E-03	−2.97E-03	−2.30E-04	−4.25E-04	−3.63E-05	−2.45	0.022	0.46
*Facklamia*	3.94E-03	−3.84E-03	8.05E-03	9.07E-04	0.02	2.33	0.029	0.46
*Klebsiella*	−4.58E-03	0.03	−0.01	−0.03	9.64E-04	−1.93	0.065	0.53
*Campylobacter*	−2.67E-03	−3.82E-03	1.64E-03	−1.35E-04	3.41E-03	1.91	0.069	0.53
*Prevotella*	7.73E-03	−3.88E-03	0.01	−1.29E-03	0.03	1.86	0.075	0.53
*Bacteroides*	0.04	7.88E-03	0.03	−3.86E-03	0.07	1.86	0.076	0.53
*Eremococcus*	4.44E-03	−4.58E-03	4.38E-03	−6.53E-04	9.42E-03	1.8	0.085	0.53
*Atopobium*	−4.74E-03	−3.33E-03	−1.75E-04	−3.77E-04	2.78E-05	−1.78	0.088	0.53

## Data Availability

All bioinformatics procedures including packages, analysis, and generation of results are detailed in Supplementary File 1 (for MRSA study) and Supplementary File 2 (for VRE study). Processed 16S rRNA gene sequencing and corresponding health metadata is in process to be deposited at SRA and dbGaP respectively.
